# Disturbances of Vaginal Microbiome Composition in Human Papillomavirus Infection and Cervical Carcinogenesis: A Qualitative Systematic Review

**DOI:** 10.3389/fonc.2022.941741

**Published:** 2022-07-12

**Authors:** Ming Wu, Huanrong Li, Hongfei Yu, Ye Yan, Chen Wang, Fei Teng, Aiping Fan, Fengxia Xue

**Affiliations:** ^1^ Department of Gynecology and Obstetrics, Tianjin Medical University General Hospital, Tianjin, China; ^2^ Tianjin Key Laboratory of Female Reproductive Health and Eugenic, Department of Gynecology and Obstetrics, Tianjin Medical University General Hospital, Tianjin, China

**Keywords:** HPV, human papillomavirus, cervical dysplasia, cervical cancer, microbiome, vaginal microbiota, systematic review

## Abstract

**Background:**

Emerging evidence has demonstrated a close association between perturbations in vaginal microbiota composition in women and human papillomavirus (HPV) infection, cervical lesions, and cervical cancer (Ca); however, these findings are highly heterogeneous and inconclusive.

**Aim:**

To perform a comprehensive systematic review of the global disturbance in the vaginal microbiota, specifically in women with HPV-associated cervical diseases, and to further conduct within- and across-disease comparisons.

**Method:**

Twenty-two records were identified in a systematic literature search of PubMed, Web of Science, and Embase up to February 28, 2022. We extracted microbial changes at the community (alpha and beta diversity) and taxonomic (relative abundance) levels. Within- and across-disease findings on the relative abundance of taxonomic assignments were qualitatively synthesized.

**Results:**

Generally, significantly higher alpha diversity was observed for HPV infection, cervical lesions, and/or cancer patients than in controls, and significant differences within beta diversity were observed for the overall microbial composition across samples. In within-disease comparisons, the genera *Gardnerella*, *Megasphaera*, *Prevotella*, *Peptostreptococcus*, and *Streptococcus* showed the greatest abundances with HPV infection; *Sneathia* and *Atopobium* showed inconsistent abundance with HPV infection, and *Staphylococcus* was observed in Ca. Across diseases, we find increased levels of *Streptococcus* and varying levels of *Gardnerella* were shared across HPV infections, high-grade squamous intraepithelial lesions, and Ca, whereas *Lactobacillus iners* varied depending on the HPV-related disease subtype.

**Conclusions:**

This systematic review reports that vaginal microbiome disturbances are correlated to the depletion of *Lactobacillus*, enrichment of anaerobes, and increased abundance of aerobic bacteria in HPV infection and related cervical diseases. Moreover, *L. iners* may exert either protective or pathogenic effects on different HPV-related diseases.

## 1 Introduction

Cervical cancer (Ca) remains the fourth most prevalent cancer in women worldwide, with 6,04,127 new cases just in 2020, and more than 3,41,831 deaths, accounting for nearly 8% of all female cancer-related deaths every year (1). This common infection-related neoplasm and its premalignant precursors are caused by high-risk human papillomavirus (HR-HPV) infections. HR-HPV persistence is a crucial contributor to the pathophysiology of cervical lesions and cancer, with preneoplastic lesions taking several years to develop (2). However, a major fraction of these patients undergo clinical HPV clearance within a few months, indicating that other potential cofactors may be involved in the progression of cervical carcinogeneses, such as vaginal microbiome disturbance.

Evidence has indicated a close relationship between the microbiome of the lower genital tract and gynecological diseases, such as polycystic ovary syndrome (PCOS), endometriosis, and HPV-related cervical diseases. Bacterial vaginosis-related (BV) microbes have been detected in PCOS and endometriosis in previous studies (3, 4). Epidemiological studies have identified relationships between vaginal dysbiosis and HPV-related diseases, particularly in BV, in relation to the risk factors involved in the initiation and progression of HPV infection (5–7). Advances in molecular microbiology have revealed perturbations in the vaginal microbiota composition in HPV-induced cervical diseases (8, 9), and cultivation-independent high-throughput sequencing has provided insights into the global patterns of vaginal microbiomes (10–12). Currently, a growing body of observational and interventional research has provided data for microbial characterization of the continuum of HPV-mediated cervical diseases. Three meta-analysis linked the epidemiological relationships among vaginal dysbiosis, HPV infection, and related cervical diseases, with case-control studies and observational investigations of vaginal dysbiosis-associated risk (6, 7). The remaining network meta-analysis of cross-sectional and longitudinal studies examined the risk of certain bacterial community types in relation to perturbations in the vaginal microbiota configuration (13). However, data have not been obtained to determine the amount and/or composition of vaginal microbiota that could potentially affect the progression of HPV infection to high-grade squamous intraepithelial lesions (HSIL) or carcinoma. In addition, the overlap of findings across the spectrum of HPV-related diseases has not yet been investigated.

Regarding specific taxa, the most common anaerobes were reported to be associated with HPV infection and cervical dysplasia (i.e., *G. vaginals*, *Megasphaera*, *Prevotella*, and *Sneathia*) (14), whereas *Sneathia* and *Atopobium* were found to be inconsistently abundant (15, 16). Other members (i.e., *Streptococcus*, *Staphylococcus*, *Corynebacterium*, *Clostridium*) have also been reported to be associated with cervical lesions and Ca (17–19). Additionally, the presence of cervical lesions and Ca among women has been correlated with an increase in *Lactobacillus iners* (13, 20) and a decrease in *Lactobacillus jensenii* and *Lactobacillus crispatus (* 20). However, conflicting results have been obtained in terms of study- and method-related heterogeneity.

Therefore, we conducted an updated systematic review of vaginal microbiome studies to characterize the microbial disturbances in women with HPV infection, HPV persistence, cervical lesions, and cervical cancer. This review aims to synthesize the findings of previous studies and conduct comparisons between- and across-diseases.

## 2 Methods

The present review was performed in accordance with the Preferred Reporting Items for Systematic Reviews and Meta-Analyses (PRISMA) statement.

### 2.1 Literature Research

A systematic literature search of PubMed, Web of Science, and Embase was carried out for articles published between January 01, 2000, and February 28, 2022. The keywords included HPV [all fields], HPV infection [all fields], human papillomavirus [all fields], cervical intraepithelial neoplasia (CIN) [all fields], cervical lesion [all fields], cervical dysplasia [all fields], cervical neoplasm [all fields], cervical cancer [all fields], cervical carcinoma [all fields], microbiome [all fields], microbiota [all fields], and flora [all fields].

### 2.2 Inclusion and Exclusion Criteria

The inclusion criteria for the study, were as follows (1): all observational studies (including case-control, cross-sectional, prospective, and retrospective cohort studies or interventional studies) (2); women with HPV infection (HPV+), HPV persistence, low-grade squamous intraepithelial lesions (LSIL), HSIL, and Ca – categorized according to the results of HPV testing and histology of cervical biopsy, compared to healthy controls (HPV-negative and cytology-negative women) or HPV-negative women (HPV-), cervical cytology was used for classification if histology was not reported (3); studies reporting microbiota analyses of vaginal samples based on high-throughput sequencing (4); studies with information on vaginal microbial alterations at the community level (alpha and beta diversity) and/or taxonomic levels (relative abundance of different microbial species).

Records were excluded if (1) they did not use high-throughput sequencing approaches (2), sequenced the microbiota from cervical or cervicovaginal swabs [because of anatomy-associated discrepancies in the microbiome (21)] (3), analyzed the microbiome in intestinal samples (4), were duplicates (5), were not published in English, or were review articles or conference abstracts.

Prospective or interventional studies reporting altered microbial composition without relevant baseline measurements were also excluded.

### 2.3 Data Extraction

Study characteristics and microbiome quantification methods were extracted from the included references. Study characteristics include the following details: publication information, subject demographics, and clinical features, whereas the latter consists of a sequencing platform, sequencing target, DNA extraction, data analysis pipelines, and reference databases. As major outcomes of the analysis, we extracted microbial alterations at the community level (alpha and beta diversity) as well as taxonomic levels (relative abundance of different microbial species). Within- and across-disease findings for the relative abundance of taxonomic assignments were qualitatively synthesized and categorized as increased, decreased, or inconsistent abundancies. No findings were consistent, with less than 75% agreement between studies reporting this taxon (22).

### 2.4 Quality Evaluation

We utilized the National Institutes of Health (NIH) National Health, Lung, and Blood Institute Study Quality Assessment Tool for Observational and Cross-sectional Studies to evaluate the internal validity and potential bias of the included studies. Study quality was rated as “good”, “fair, or poor” by two authors (MW and HL), with differences addressed through discussion.

## 3 Results

### 3.1 Search Results

**Figure 1:**
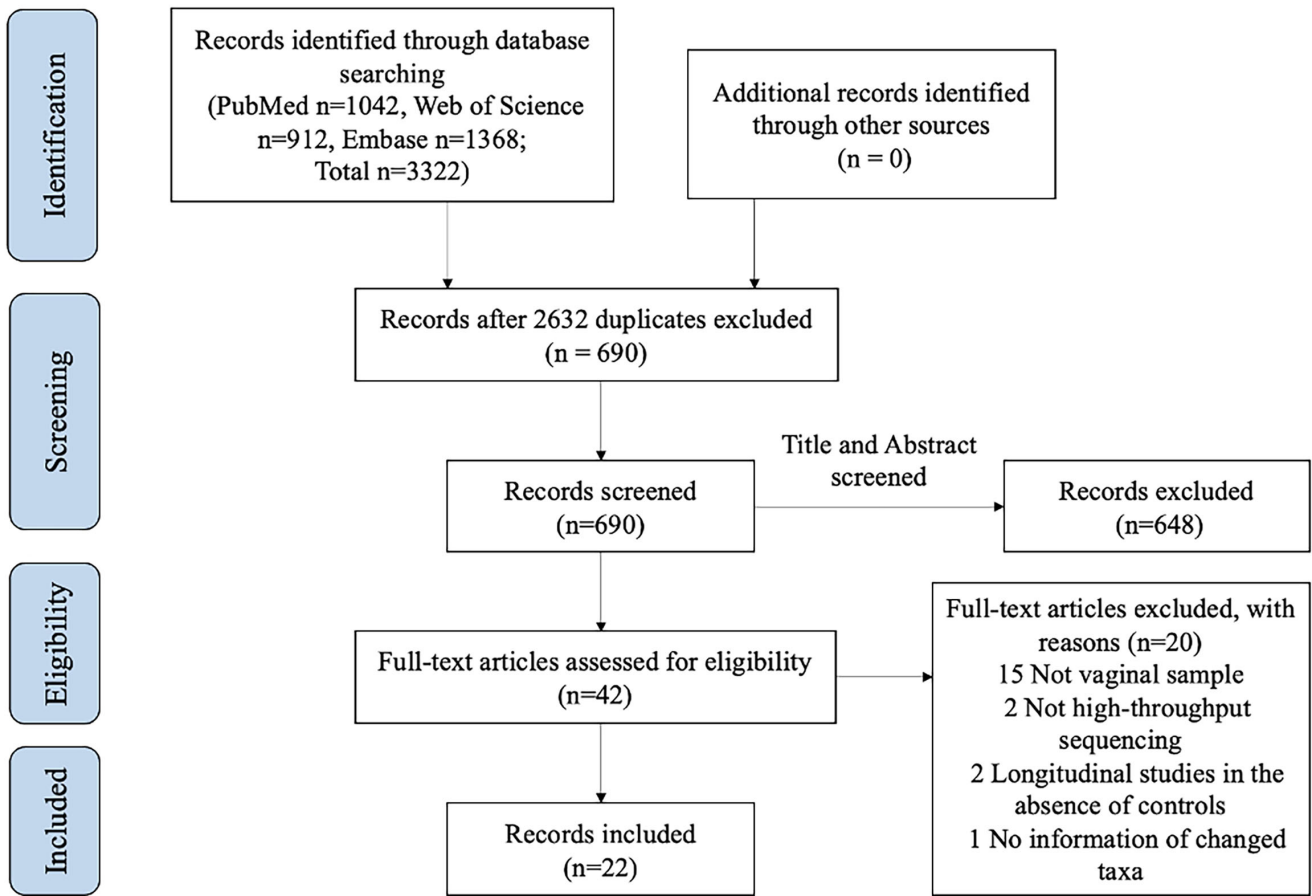
PRISMA flow diagram of the study selection process for inclusion.

A total of 3322 relevant records were obtained in the initial electronic search, including 1042 from PubMed, 912 from Web of Science, and 1368 from Embase. Six hundred ninety-nine studies were imported after removing duplicates and screening the titles and/or abstracts. Of those, studies were excluded due to non-vaginal samples (n=15), non-high-throughput sequencing approaches (n=2), no information on any change in taxa compared with HPV- (n=1), and longitudinal studies in the absence of controls (n=2) after assessing the full text (n=42). Ultimately, 22 studies were selected for the final analysis. A flowchart of this process is shown in **Figure 1**.

### 3.2 Characteristics of the Included Studies

The 22 studies consisted of 17 case-control, three cross-sectional, and two longitudinal studies. Most studies (72.7%, 16/22) were conducted in Asia (China, Korea), five (22.7%) were performed in Western countries (USA, UK, Mexico, and Sweden), and one (4.5%) was performed in Africa (Nigeria). In the 17 case-control comparisons, six studies compared HPV+ or HPV persistence to the controls, ten studies compared women with cervical lesions and/or Ca to the controls, and 3 included women with HPV infection. Only one study compared ≥ HSIL with LSIL. Of these, one study grouped samples into LSIL, HSIL, and normal cytology groups based on observed cytology. In the two prospective studies, comparisons were performed between cases before treatment (local excisional treatment and neoadjuvant chemotherapy) and untreated controls. The detailed information is provided in **Table 1**.

**Table 1:** Characteristics of the included studies.

Study type	Country	Study	Year	Sub-groups (N)	Controls (N)	HPV types (N)	Age (year)	Sample collection (site)
Case-control	England	Mitra et al. (23)	2015	LSIL (52) HSIL (92) Ca (5)	20	HPV+: 93	18-45	posterior vaginal fornix
Case-control	USA	Laniewski et al. (24)	2018	HPV+ (31) LSIL (12) HSIL (27) Ca (10)	20	HR-HPV+: 73 LR-HPV+: 7	Control: 39.55 ± 7.35 HPV+: 37.64 ± 9.38 LSIL: 35.08 ± 7.24 HSIL: 38.29 ± 8.46 Ca: 38.90 ± 9.09	vaginal swabs
Case-control	China	Chao et al. (16)	2019	HPV+ (65)	86	HR-HPV+: 65	20-65	posterior vaginal fornix
Case-control	China	Yang et al. (25)	2020	HPV16 + (25)	27	HPV16 +.: 25	25-50	near the vaginal fornix and cervix
Case-control	China	Liu et al. (26)	2020	HPV persistence (48) HPV transience (43)	31	NR	NR	vaginal secretions
Case-control	China	Chao et al. (27)	2020	HPV persistence (59) HPV transience (139)	131	HPV16/18 +.: 79 HPV others +: 121 NA: 7	20-69	posterior vaginal fornix
Case-control	China	Chen et al. (28)	2020	HPV+ (78) LSIL (51) HSIL (23) Ca (9)	68	HPV16/18 +: 22 HPV other 12 +: 36 LR-HPV+: 92	25-69	lateral and posterior fornix
Case-control	China	Cheng et al. (29)	2020	LSIL (26) HSIL (40) Ca (32)	33	HR-HPV+: 98	21-65	vaginal fornix or the middle side of the vagina
Case-control	Korea	Lee et al. (30)	2020	≤LSIL (24) ≥HSIL (42)	–	HPV+: 48 HPV16/18 +: 24	45.1 ± 11.7	posterior vaginal fornix
Case-control	China	Xie et al. (31)	2020	CIN (30) Ca (30)	30	HPV16/18 +: 32 HPV others +: 10 HPV-: 3	25-39	posterior vaginal fornix
Case-control	China	Wu et al. (32)	2020	LSIL^a^ (22) HSIL^a^ (16)	31 (NILM)	HPV+:21 HR-HPV: 34 HPV-: 14	16-50	posterior vaginal fornix
Case-control	China	Wei et al. (15)	2021	HPV+ (30)	30	HR-HPV+: 30	20-49	mid-vagina
Case-control	Mexico	Nieves-Ramírez et al. (33)	2021	LSIL (90) HSIL (31)	107	HPV+: 156 HPV-: 72	all: ≥ 21 sil:37.26 ± 10.87 normal:42.83± 7.92	vaginal exudate
Case-control	Korea	Kang et al. (34)	2021	HSIL (8) Ca (8)	7	HPV+: 15 HPV16/18 +: 5	Controls: 47.4 ± 5.38 HSIL: 43.4 ± 12.8 Ca: 47 ± 10.2	vaginal swabs
Case-control	China	Chao et al. (35)	2021	HPV+ (86) HSIL (83)	103	HPV16/18 +: 63 HPV others +: 90	20-72	posterior vaginal fornix
Case-control	China	Fan et al. (19)	2021	Ca (65)	54 (HPV+: 47)	HPV+: 63 HPV-: 2	Ca: 48.65 ± 6.873 Controls: 46.81 ± 8.15	vaginal samples
Case-control	China	Mei et al. (36)	2022	HPV persistence (28) HPV clearance (30)	42	HR-HPV+: 58 HR-HPV-: 42	21-64	mid-vaginal secretion samples
cross-sectional	Nigeria	Dareng et al. (37)	2016	278		HR-HPV+: 66 (HIV +: 53, 81.5%) HR-HPV-:212 (HIV +: 98 (49.7%)	≥ 18	mid-vaginal
cross-sectional	Sweden	Cheng et al. (38)	2020	257		HPV+: 144 HPV-: 113	14-29	vaginal swabs
cross-sectional	China	Lin et al. (39)	2022	448 (sub-samples: 23 HPV+ vs 5 HPV-)		HR-HPV+: 164 HR-HPV-: 34	20-74	upper third of vaginal walls
Longitudinal (prior to local excision)	UK	Mitra et al. (40)	2021	LSIL (15) HSIL (88)	39 (NILM)	NA	18-45	vaginal swab
Longitudinal (prior to neoadjuvant chemotherapy)	China	Wang et al. (18)	2021	Ca (26)	40	HPV16/18 +: 17 HPV others +: 1 HPV-: 8	Ca: 53.38 (48.00~58.75) Controls: 50.00 (44~54.50)	vaginal samples

a classified by cervical cytology; NILM, negative for intraepithelial lesion or malignancy; LSIL, low-grade squamous intraepithelial lesions; HSIL, high-grade squamous intraepithelial lesions; Ca, cervical cancer; HPV, human papillomavirus; HR-HPV, high-risk HPV infection; LR-HPV, low-risk HPV infection; HPV+, HPV-positive women; HPV-, HPV-negative women. NA, not available; NR, not reported.

Control samples were defined as healthy women in most studies, whereas in two studies the controls were defined as having normal cytology. The controls were HPV positive in one study.

### 3.3 Methodological Summary

The different microbiome analysis methods are shown in **Table 2**. Sequencing approaches included 16S rRNA (95.5%, 21/22) and metagenomics (4.5%, 1/22). Among the studies employing 16S sequencing, sequencing was predominantly performed using the Illumina MiSeq platform (52.4%, 11/21), followed by the HiSeq platform (33.3%, 7/21), the Ion Torrent PGM platform (9.5%, 2/21), and the Novaseq platform (4.8%, 1/21). Five different hypervariable regions were amplified, including V1-V2 (4.8%, 1/21), V1-V3 (4.8%, 1/21), V3 (9.5%, 2/21), V4 (42.9%, 9/21), and V3-V4 (33.3%, 7/21). However, one study did not provide a sequencing target. The bioinformatics pipelines used included QIIME (52.4%, 11/21), Mothur (19.0%, 4/21), QIIME and DATA2 (4.8%, 1/21), DATA2 (4.8%, 1/21), and USEARCH (4.8%, 1/21). The widely used reference databases for the taxonomic assignment were the SILVA (33.3%, 7/21), Greengenes (23.8%, 5/21), and Ribosomal Database Project (14.3%, 3/21) databases.

**Table 2:** Microbiome methodology of included studies.

Study	Year	Sequencing platform	Analysis technique	Sequencing target	Temperature for Storage	DNA Extraction	Data analysis pipelines	Taxonomic assignment database
Mitra et al. (23)	2015	Illumina MiSeq	16S rRNA gene sequencing	V1-2	−80 °C	QiAmp Mini DNA kit (Qiagen, Venlo, Netherlands)	Mothur	RDP
Laniewski et al. (24)	2018	Illumina MiSeq	16S rRNA gene sequencing	V4	−80 °C	PowerSoil DNA Isolation Kit (MO BIO Laboratories, Carlsbad, CA)	USEARCH	Greengenes
Chao et al. (16)	2019	Illumina HiSeq 2500	16S rRNA gene sequencing	V4	−80 °C	NR	QIIME	NR
Yang et al. (25)	2020	Illumina Hiseq X-ten	shotgun metagenomic	–	−80 °C	Qiagen QIAmp DNA Microbiome Kit (Qiagen)	SOAPdenovo	edicted open reading frames (ORFs) wer
Liu et al. (26)	2020	Illumina HiSeq 2500	16S rRNA amplicon pyrosequencing	NR	NR	MOBIO PowerFecal DNA isolation kit	QIIME 2	Greengenes
Chao et al. (27)	2020	Illumina HiSeq 2500	16S rRNA gene sequencing	V4	−80 °C	TruSeq ^®^ DNA PCR-Free Sample Preparation Kit (Illumina, USA)	QIIME	SILVA
Chen et al. (28)	2020	Illumina MiSeq	16S rRNA gene sequencing	V3-4	−80 °C	QIAamp DNA Mini Kit (Qiagen^®^)	QIIME	RDP
Cheng et al.	2020	Illumina HiSeq 2000	16S rRNA gene sequencing	V4	NR	Genomic DNA kits (Beijing Bioteke, China)	NR	NR
Lee et al. (30)	2020	Ion Torrent PGM	16S rRNA gene sequencing	V3	−80 °C	QIAamp PowerFecal Pro DNA Kit (QIAGEN, Germany)	QIIME	Greengenes
Xie et al. (31)	2020	Illumina Novaseq	16S rDNA gene sequencing	V4	−80 °C	Tiangen Biotech Co., Ltd., Beijing, China	QIIME	NR
Study	Year	Sequencing platform	Analysis technique	Sequencing target	Temperature for Storage	DNA Extraction	Data analysis pipelines	Taxonomic assignment database
Wu et al. (32)	2020	Illumina MiSeq	16S rRNA gene sequencing	V3-4	−80 °C	NR	NR	NR
Wei et al.	2021	Illumina MiSeq PE300	16S rRNA gene sequencing	V3-4	−80 °C	E.Z.N.A. ^®^RDNA extraction kits (Omega BioTek, Norcross, GA, USA)	QIIME	Silva and UCLUST software
Nieves-Ramírez et al.	2021	Illumina HiSeq 2000	16S rRNA gene sequencing	V3	−20 °C	FastDNA spin kit for soil (MP Biomedicals)	Mothur	SILVA
Kang et al. (34)	2021	Ion Torrent PGM	16S rRNA gene sequencing	V4	NR	QIAamp PowerSoil Pro DNA Kit (QIAGEN, Hilden, Germany)	QIIME2 and DADA2	SILVA
Chao et al. (35)	2021	Illumina HiSeq 2500	16S rRNA gene sequencing	V4	−80 °C	NR	NR	NR
Fan et al. (19)	2021	Illumina HiSeq	16S rRNA gene sequencing	V3-4	−80 °C	QlAamp DNA mini Kit (Qiagen)	QIIME	NR
Mei et al. (36)	2022	Illumina MiSeq	16S rRNA gene sequencing	V3-4	NR	AxyPrep DNA Gel Extraction Kit (Axygen Biosciences, Union City, CA, USA)	NR	Silva
Dareng et al. (37)	2016	Illumina MiSeq	16S rRNA gene sequencing	V4	NR	NR	QIIME	Greengenes
Cheng et al.	2020	Illumina MiSeq	16S rRNA gene sequencing	V3-4	NR	ZR-96 Genomic DNA MagPrep kit (Zymo Research, USA)	DADA2	SILVA
Lin et al. (39)	2022	Illumina MiSeq	16S rRNA gene sequencing	V4	−20 °C	E.Z.N.A Mag-Bind Soil DNA Kit (Omega Bio-Tek, GA, USA)	Mothur	SILVA
Mitra et al. (40)	2021	Illumina MiSeq	16S rRNA gene sequencing	V1-3	−80 °C	QIAmp DNA mini kit (Qiagen, Venlo, The Netherlands)	Mothur	RDP and STIRRUPS
Wang et al. (18)	2021	Illumina MiSeq	16S rRNA gene sequencing	V3-4	−80 °C	NR	QIIME	NR

NR, not reported; RDP, Ribosomal database project.

### 3.4 Alpha Diversity and Beta Diversity

**Figure 2:**
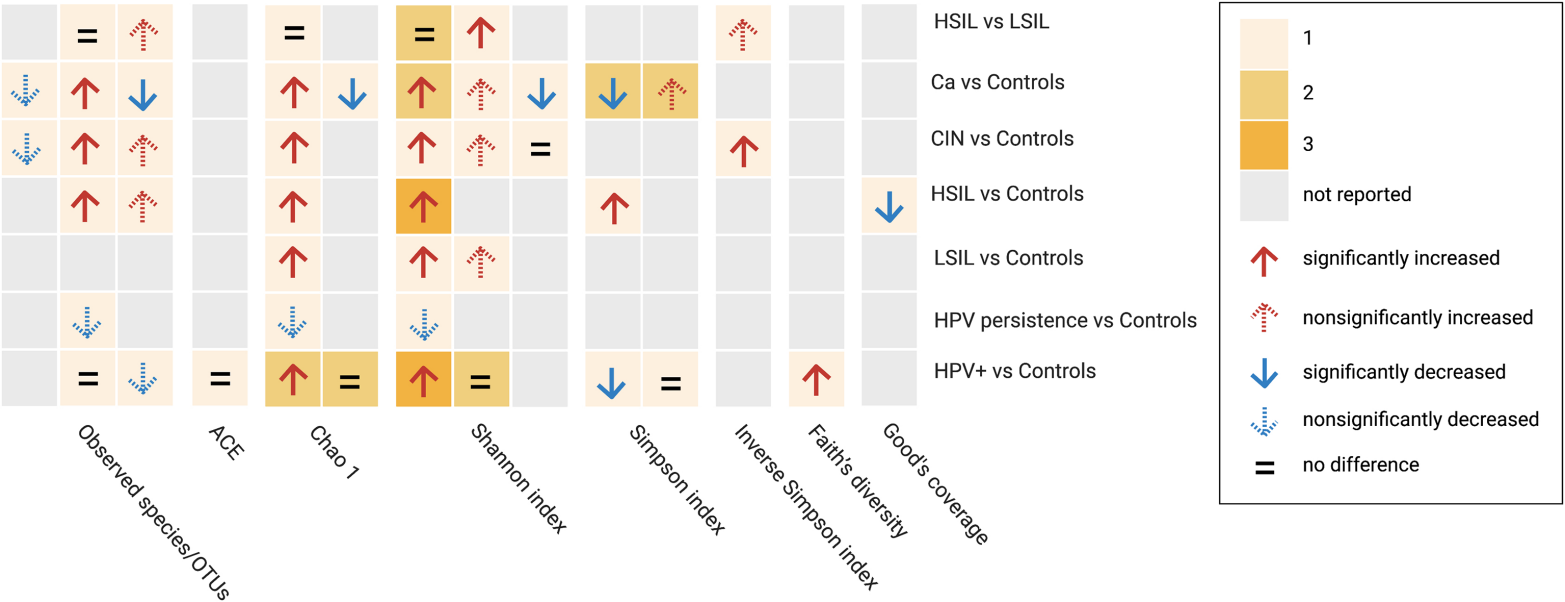
Alpha diversity assessments reported by studies between subgroup and controls based on different metrics. Cells are colored based on the number of assessments. HPV+, HPV-positive women; LSIL, low-grade squamous intraepithelial lesions; HSIL; high-grade squamous intraepithelial lesions; CIN, cervical intraepithelial neoplasia; Ca, cervical cancer; OTUs, operational taxonimic units.

A total of 57 alpha-diversity assessments were performed across 21 studies (**Figure 2**) Reported metrics included measurements of richness (observed species/operational taxonomic units, Chao1, abundance-based coverage estimator; 43.9%, 25/57), richness and evenness (Shannon, Simpson, inverse Simpson; 51.7%, 30/58), biodiversity (Faith’s phylogenetic diversity, 1.8%, 1/57) and sequencing depth (Good’s coverage, 1.8%, 1/57). Most of the reported metrics were based on the Shannon index (36.8%, 21/57).

When comparing HPV+ vs. controls or HPV−, five studies examined community richness using different metrics. Two studies indicated that there were no significant differences, two found significantly higher richness indices, whereas one found a non-significant decrease in richness indices. Regarding diversity, combining richness and evenness, significantly higher diversity was observed in three studies, significantly lower diversity was observed in one study, and no significant differences were observed in three studies. One study found a significant increase in Faith’s phylogenetic diversity.

When comparing HPV persistence vs. controls, lower richness indices were reported in two studies, and lower alpha diversity based on a combination of richness and evenness data was obtained in one study.

In comparisons between CIN and controls, two studies investigated higher richness and diversity by incorporating richness evenness indices in LSIL, four studies reported increases in both metrics in HSIL, and four studies reported a similar pattern in CIN. Among women with Ca, 4 studies reported differential richness data. Higher richness was reported in 2 studies, and lower richness was reported in the remaining studies. Differential diversity indices combining richness and evenness were reported in five studies, four of which reported higher diversity and two of which reported lower diversity. Four studies examined diversity based on a comparison between HSIL and LSIL, two of which reported no significant observations in terms of richness and/or evenness data, with the remaining studies providing higher indices. In addition, a significantly lower Good’s coverage was reported in one study comparing HSIL and controls.

Beta diversity comparisons were evaluated in 15 studies, with 13 studies comparing subgroup vs. controls, one study comparing combined subgroups vs. controls, and one study comparing within-subgroup results. Six of 13 studies identified significant differences between different samples, with 4 of 13 showing visually separated components with no significance, 2 finding no clear separation between groups, and 1 study without a report. In the across-group (controls excluded) comparisons, two studies reported no significant differences between subgroups (≥ HSIL vs. ≤ LSIL, HSIL vs. LSIL, and CIN vs. HPV+). One subgroup (HPV+ + LSIL + Ca) was significantly different from the controls. This study also showed a significant difference between the combined subgroup (LSIL + HSIL) and HPV infections. The beta diversity assessments are presented in **Table 3**.

**Table 3:** Beta diversity reported by studies.

Study	Subgroup vs controls	Beta diversity		Findings	*P-*value
		Metric	Analysis		
Chao et al., 2019 (16)	HPV+ vs control	Unweighted UniFrac		sig. different	*P* < 0.05
Chen et al., 2020 (28)	HPV+ vs control	Unweighted UniFrac	PCA	visually separated	NR
Wei et al., 2021 (15)	HPV+ vs control	NR	PCA	slightly different	NR
Cheng et al., 2020 (38)	HPV+ vs HPV-	Bray-Curtis UniFrac phylogenetic	PCoA	no clear separation	NR
Wang et al., 2021 (18)	Ca vs control	Bray-Curtis	PCoA	sig. different	*P* < 0.01
Fan et al., 2021 (19)	Ca vs control	NR	PCA	sig. different	NR
Mei et al., 2022 (36)	within the persistent, clearance, control groups	Bray-Curtis	PCoA	sig. different	*P* = 0.001
Chao et al., 2020 (27)	within the HPV persistence, HPV transience and control groups	Unweighted UniFrac	PCA	NR	NR
Mitra et al., 2015 (23)	within the HPV+, LSIL and HSIL, Ca, controls	NR	PCA	Three major clusters corresponding to samples	NR
Laniewski et al., 2018 (24)	within the HPV+, LSIL and HSIL, Ca, controls	Bray-Curtis	PCA	No cluster	NR
Xie et al., 2020 (31)	within the CIN, Ca and controls	Bray-Curtis	PCoA	Dots in controls obviously deviated from in CIN and Ca	NR
Kang et al., 2021 (34)	within the HSIL, Ca and control groups	Bray-Curtis	PCoA	sig. different	*P* = 0.001
Study	Subgroup vs controls	Beta diversity		Findings	p-value
		Metric	Analysis		
Lee et al., 2020 (30)	≥ HSIL vs ≤ LSIL	NMDS	PCA	no sig. difference	*P* = 0.443
Nieves-Ramírez et al. 2021 (33)	SIL vs women without HPV infection or SIL; LSIL vs HSIL; HPV+ with CIN vs HPV+ without CIN	Bray-Curtis	PCoA	sig. different; no sig. difference; no sig. difference	*P* = 0.002
Chen et al., 2020 (28)	HPV+ + LSIL + Ca vs control; LSIL + HSIL vs HPV+	Unweighted UniFrac	PCA	sig. different; sig. different	*P* < 0.05

NR, not reported; LSIL, low-grade squamous intraepithelial lesions; HSIL, high-grade squamous intraepithelial lesions; Ca, cervical cancer; HPV, human papillomavirus; HPV+, HPV-positive women; HPV-, HPV-negative women; NMDS, non-metric multi-dimensional scaling; PCoA, principal coordinateanalysis; PCA, principal component analysis; sig., significantly; no sig., non significantly.Quality Assessment of Reviewed Studies.

### 3.5 Taxonomic Findings

Twenty-one studies have reported the relative abundance of vaginal microorganisms at the phylum, family, genus, and species levels. Between-subgroup comparisons of CIN were conducted relative to controls or in the order of abundance in subgroups reported by previous studies.

**Figure 3:**
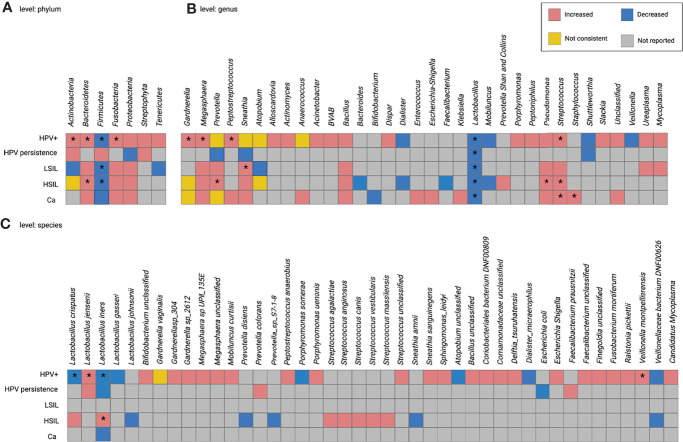
Within- and cross-disease changes of relative abundance of microbial taxa. *The relative abundance of taxa reported by more than 1 study. Not consistent, any finding with less than 75% agreement between studies reporting this taxon. HPV+, HPV-positive women; LSIL, low-grade squamous intraepithelial lesions; HSIL; high-grade squamous intraepithelial lesions; CIN, cervical intraepithelial neoplasia; Ca, cervical cancer.

**Figure 2** presents a summary of within- and across-disease comparisons for HPV-related diseases at the phylum, genus, and species levels. Taxa reported as increased or decreased in the subgroups of each study were combined. High inconsistencies were observed at different taxonomic levels in both comparisons. The following results were obtained for the taxa provided by at least two studies:

(1)In HPV+, the abundance of thirteen taxa increased or decreased: the nine with increased abundancies included the phyla *Actinobacteria*, *Bacteroidetes*, and *Fusobacteria*; the genera *Gardnerella*, *Megasphaera*, *Peptostreptococcus*, and *Streptococcus*; the species *L. jensenii* and *Veillonella montpellierensis*); the four taxa with a decrease in abundance included the phylum *Firmicutes*, the genus *Lactobacillus*, and the species *L. crispatus* and *L. iners*). *Prevotella*, *Sneathia*, *Atopobium*, *Anaerococcus*, and *Gardnerella vaginalis* were inconsistently identified (**Figure 3**).
(2)In HPV persistence, *Lactobacillus* numbers were decreased (**Figure 3**).
(3)In CIN, one increase in abundance in taxon (*Sneathia*) and two decrease in abundance in taxa (the phylum *Firmicutes* and the genus *Lactobacillus*) were observed in LSIL; five increased taxa (phylum *Bacteroidetes*, genera *Prevotella*, *Streptococcus* and *Pseudomonas*, and species *L. iners)*, two decreased taxa (phylum *Firmicutes* and genus *Lactobacillus)*, and three taxa with inconsistent numbers (phylum *Actinobacteria*, genera *Gardnerella* and *Atopobium*) were reported in HSIL (**Figure 3**).
(4)In Ca, the numbers of *Streptococcus* and *Staphylococcus* were increased, while *Lactobacillus* was decreased. *Gardnerella* and *Megasphaera* provided inconsistent data (**Figure 3**).

The differential numbers of some taxa were consistent between the subgroups. For example, higher *Bacteroidetes* was common to both HPV+ and HSIL; lower *Firmicutes* was common to HPV+, LSIL, and HSIL; higher *Streptococcus* was common to HPV+, HSIL, and Ca; lower *Lactobacillus* was common to all subgroups. The genera *Gardnerella* and *Prevotella* were inconsistently present in HPV+, HSIL, and Ca; *Sneathia* was inconsistent in HPV+ and LSIL; *L. iners* was inconsistent in HPV+ and HSIL (**Figure 3**).

### 3.6 Quality Assessment

Most studies were rated ‘fair’ (63.7%, 14/22). Only four studies were rated as ‘Good’ (18.2%, 4/22), which not only included study features, microbiome methods, and rich reporting of major outcomes but also considered several potential confounders in the reports. Four studies were rated as ‘poor’ quality (18.2%, 4/22). The major sources of potential bias were the lack of reporting of tools or methods for measuring outcomes, lack of detailed information on demographic or clinical characteristics, and lack of detailed definition of cases. The internal validity of the included studies is shown in **Table 4**.

**Table 4:** Quality assessment of included studies using the Quality Assessment Tool for Observational Cohort and Cross-Sectional Studies.

Study	Q1	Q2	Q3	Q4	Q5	Q6	Q7	Q8	Q9	Q10	Q11	Q12	Q13	Q14	Quality Rating	Notes (if poor)
Mitra et al., 2015 (23)	Yes	Yes	Yes	Yes	Yes	No	No	Yes	Yes	NA	Yes	NA	NA	Yes	Good	
Laniewski et al., 2018 (24)	Yes	Yes	Yes	Yes	Yes	No	No	Yes	Yes	NA	Yes	NA	NA	Yes	Good	
Chao et al., 2019 (16)	Yes	Yes	Yes	Yes	Yes	No	No	NA	Yes	NA	CD	NA	NA	No	Fair	
Yang et al., 2020 (25)	Yes	Yes	Yes	Yes	Yes	No	No	NA	Yes	NA	Yes	NA	NA	No	Fair	
Liu et al., 2020 (26)	Yes	No	Yes	CD	No	No	No	Yes	Yes	NA	CD	NA	NA	No	Poor	The information of demographic and clinical features of subjects are not provided, such as age and HPV subtypes, and it does not define major outcomes or power description clearly. It is unclear that whether the subjects were selected from the same or similar populations. The sequencing target for amplicon is not reported.
Chao et al., 2020 (27)	Yes	Yes	Yes	Yes	Yes	No	No	Yes	Yes	NA	Yes	NA	NA	No	Fair	
Chen et al., 2020 (28)	Yes	Yes	Yes	Yes	Yes	No	No	Yes	Yes	NA	Yes	NA	NA	Yes	Good	
Cheng et al., 2020	Yes	Yes	Yes	Yes	Yes	No	No	Yes	Yes	NA	CD	NA	NA	No	Poor	The tools or methods for measuring outcomes, including analysis pipelines and reference database are not provided. It is also unclear to provide the information of microbial composition in detail, including beta diversity. The study reports that “A total of 131 women who were examined or diagnosed in our hospital from September 2016 to June 2019 were selected.” indicating the likelihood of substantial temporal delay.
Lee et al., 2020 (30)	Yes	Yes	Yes	CD	Yes	No	No	Yes	Yes	NA	Yes	NA	NA	No	Fair	
Xie et al., 2020 (31)	Yes	No	Yes	Yes	Yes	No	No	Yes	Yes	NA	CD	NA	NA	No	Poor	The tool or method viz, reference database for assigning species is not reported. The women with CIN were not defined in detail, thereby complicating the synthesis of findings.
Wu et al., 2020 (32)	Yes	Yes	Yes	Yes	Yes	No	No	Yes	Yes	NA	CD	NA	NA	No	Poor	The tools or methods, such as DNA extraction kit, analysis pipeline and reference database are provided. And the population are categorized by cervical cytology, thus complicating the synthesis of findings.
Wei et al., 2021	Yes	Yes	Yes	Yes	Yes	No	No	NA	Yes	NA	Yes	NA	NA	No	Fair	
Nieves-Ramírez et al., 2021 (33)	Yes	Yes	Yes	Yes	Yes	No	No	Yes	Yes	NA	Yes	NA	NA	Yes	Good	
Kang et al., 2021 (34)	Yes	Yes	Yes	CD	Yes	No	No	Yes	Yes	NA	Yes	NA	NA	No	Fair	
Chao et al., 2021 (35)	Yes	Yes	Yes	Yes	Yes	No	No	Yes	Yes	NA	CD	NA	NA	No	Fair	
Fan et al., 2021 (19)	Yes	Yes	Yes	CD	Yes	No	No	NA	Yes	NA	CD	NA	NA	No	Fair	
Mei et al., 2022 (36)	Yes	Yes	Yes	Yes	Yes	No	No	Yes	Yes	NA	CD	NA	NA	No	Fair	
Dareng et al., 2016 (37)	Yes	Yes	Yes	Yes	Yes	No	No	NA	Yes	NA	Yes	NA	NA	Yes	Fair	
Cheng et al., 2020	Yes	Yes	Yes	Yes	Yes	No	No	NA	Yes	NA	Yes	NA	NA	Yes	Fair	
Lin et al., 2022 (39)	Yes	Yes	Yes	Yes	Yes	No	No	NA	No	NA	Yes	NA	NA	No	Fair	
Mitra et al., 2021 (40)	Yes	Yes	Yes	Yes	Yes	No	No	Yes	Yes	NA	Yes	NA	NA	No	Fair	
Wang et al., 2021 (18)	Yes	Yes	Yes	CD	Yes	No	No	NA	Yes	NA	CD	NA	NA	No	Fair	

National Institutes of Health National Heart, Lung and Blood Institute Study Quality Assessment Tool for Observational Cohort and Cross-sectional Studies. NA, not applicable.

Q1. Was the research question or objective in this paper clearly stated?

Q2. Was the study population clearly specified and defined?

Q3. Was the participation rate of eligible persons at least 50%xx?

Q4. Were all the subjects selected or recruited from the same or similar populations (including the same time period)? Were inclusion and exclusion criteria for being in the study prespecified and applied uniformly to all participants?

Q5. Was a sample size justification, power description, or variance and effect estimates provided?

Q6. For the analyses in this paper, were the exposure(s) of interest measured prior to the outcome(s) being measured?

Q7. Was the timeframe sufficient so that one could reasonably expect to see an association between exposure and outcome if it existed?

Q8. For exposures that can vary in amount or level, did the study examine different levels of the exposure as related to the outcome (e.g., categories of exposure, or exposure measured as continuous variable)?

Q9. Were the exposure measures (independent variables) clearly defined, valid, reliable, and implemented consistently across all study participants?

Q10. Was the exposure(s) assessed more than once over time?

Q11. Were the outcome measures (dependent variables) clearly defined, valid, reliable, and implemented consistently across all study participants?

Q12. Were the outcome assessors blinded to the exposure status of participants?

Q13. Was loss to follow-up after baseline 20% or less?

Q14. Were key potential confounding variables measured and adjusted statistically for their impact on the relationship between exposure(s) and outcome(s)?

## 4 Discussion

Our systematic review evaluated vaginal microbiome disturbances across a spectrum of HPV-associated diseases to analyze compositional alterations and perform within- and across-disease comparisons. A total of 22 studies were included in the meta-analysis. First, we outline the variances in both the study features and methodology. Next, a qualitative synthesis indicated that the alpha diversity was significantly higher than that of the controls, whereas the beta diversity showed a significant difference in the overall microbial composition across samples. The patterns of observed taxonomic changes revealed a large heterogeneity at the within- and across-disease levels. When considering alterations in abundance in at least two studies, we analyzed the alterations in consistent and inconsistent directions, within the disease. In addition, our results revealed an overlap across diseases in consistently and inconsistently altered taxa. The underlying mechanisms explaining the observed disturbances are discussed for both comparisons. The confounding effects of study characteristics and microbiome methodologies were also assessed.

### 4.1 Diversity

Overall, our synthesis indicated a significant increase in richness and diversity in women with cervical lesions and cancer, but no difference was observed between HSIL and LSIL. Medium and large studies indicated a non-significant relationship between richness and/or evenness indices in women with HPV infection, suggesting that alpha diversity indices are of limited utility as a measurement of vaginal health, or for distinguishing between HPV infection and controls. A large number of intrinsic and extrinsic factors account for variations in diversity at the baseline, including race, age, menstrual cycles, sexual activities, contraception use, smoking, and diet, thereby complicating the measurement (33, 41–46).

However, reports with inconsistent data have been published. A few studies have reported a reduction in the richness and diversity of HPV infections. A similar pattern was observed in women with HPV persistence and Ca. These residual heterogeneities for certain diseases may be attributed to methodological effects and the study design. Alpha diversity is particularly affected by measurement errors in microbiome research, owing to the heavy bias of commonly used estimators (47). Although the number of sequences affects the alpha diversity estimates, several confounders in combination with data pre-processing may influence the data output (47, 48). Furthermore, richness metrics do not account for unobserved taxa or provide variance estimates (47). These multifactorial aspects may have contributed to the heterogeneous findings. It is worth noting that in one study investigating lower alpha diversity in Ca, a higher proportion of HPV+ women were a part of the control cohort, which may have resulted in less harmonization in the synthesis.

Regarding beta diversity, most studies have observed a significant cluster between the samples. In summary, two of the studies reported no significant differences between HSIL and LSIL, which is consistent with the pattern observed for alpha diversity.

### 4.2 Alternations in Vaginal Microbiome Composition

#### 4.2.1 Within-Disease Comparisons

Generally, when considering the differential taxa provided by more than two studies, we found that specific alterations occurred within the disease at the genus and species levels. The genus *Staphylococcus* was the only taxon enriched in Ca whereas, the genera *Gardnerella*, *Megasphaera*, *Prevotella*, *Peptostreptococcus*, and *Streptococcus* showed the most common increase in HPV infections, most of which were BV-related anaerobes, although they were also reported in HSIL and Ca. These associations are likely mediated by biological amine-induced oxidative stress since BV-related bacteria are linked to their production (14). Recent studies have highlighted the complex and bi-directional association between HPV and BV. Mechanistically, HPV inhibits basal and proinflammatory-induced host defense peptide expression by subverting the NF-κB and Wnt/β-catenin signaling cascades, thus leading to a significant reduction in the *Lactobacillus* species that rely on amino acid sources, which further promotes an imbalance in vaginal flora. In turn, oxidative stress resulting from BV infiltration promotes the progression of preneoplastic lesions (49).

Intriguingly, the genera *Sneathia* and *Atopobium*, two important, yet underappreciated pathogens, play controversial roles in HPV acquisition. *Sneathia* is the only microorganism enriched in the initiation and progression of cervical carcinogenesis (24) and arises as a consequence of the disease (14). Our analysis recapitulated that overrepresentation of *Sneathia* was reported in three studies comparing HPV infection and controls. Two studies reported depletion, one of which used only 16S rRNA amplicon pyrosequencing. *Atopobium* showed findings similar to those of our review. A plausible contribution to the pathomechanism may indicate the potential release of toxic products by adherent *Sneathia* to alter the characteristics of host tissue and directly mediate the effects on the cervical microenvironment (50). It is postulated that the enzyme, sialidase, facilitates the destruction of the mucus layer on the vaginal epithelium and entraps *Atopobium* (28). In addition, *Prevotella bivia*, as an early colonizer in BV, may pave the way for secondary colonizers like *Atopobium* and *Sneathia* (51). In contrast, a viral-driven over-reactive host immune response may lead to an observed decrease in the two pro-inflammatory genera (15). These findings represent a plausible mechanism to explain the contribution of *Sneathia* and *Atopobium* to the initiation of HPV infection. It is noteworthy that few studies have provided inconsistent abundance data on taxa, although such results may be more reflective of methodological aspects, rather than true intraspecific diversity.

#### 4.2.2 Across-Disease Comparisons

Importantly, our observations implied that HPV-associated diseases share similar microbial alterations. Typically, we found an overlap between HPV infection, HSIL, and Ca in consistently and inconsistently altered taxa. Specifically, the anaerobe *Gardnerella* was enriched in HPV infection. An inconsistent change in HSIL was reported in two studies, which was also observed for Ca. These associations suggest that BV-related microbes may be considered indirect markers of sexual transmission of HPV, rather than promoters of progression to more severe lesions (52). Moreover, the genus *Streptococcus* was the only enriched taxon found in HPV infection, HSIL, and Ca, suggesting that enrichment of this genus may be characteristic of HPV-associated diseases, irrespective of the subtype. As described before, the genus *Staphylococcus* was the only taxon enriched in Ca. Consistently, 12 species were found in women with Ca, mainly *Corynebacterium* spp. and *Staphylococcus* spp., based on the identification of the cultivable aerobic bacterial microbiota (17). Notably, inflammation is crucial for the progression of cervical cancer to cancer (53, 54). A dysbiotic microbiome dominated by aerobic species can create an inflammatory environment that is favorable for tissue damage. It can also drive pathology by promoting immune evasion that favors tumor cell survival (17). Apart from BV-related anaerobes, the enrichment of other genera, such as *Streptococcus* and *Staphylococcus*, should be considered in the interpretation of vaginal microbiota during cervical carcinogenesis, which has been discussed in previous studies (52, 55). Our work strengthens this hypothesis by demonstrating that specific aerobic microorganisms may exert dominant functionality that is relevant to the progression of cervical lesions. Further studies are required to investigate the contribution of aerobic bacteria to cervical carcinogenesis.

Furthermore, *L. iners*, a strain that is the most common vaginal bacterium in healthy women (56), was found to be lower in HPV infection, HPV persistence, and Ca, but higher in HSIL. For example, decreased *L. iners* numbers have been linked to HPV infection, HPV persistence, and Ca (19, 27, 28). Alternatively, the presence of pre-cancer and cancer in women is associated with a high relative abundance of *L. iners* (20). This observation was partially confirmed here since two studies implicated increased numbers of *L. iners* in HSIL (30, 35). A plausible explanation is that species in the *Lactobacillus* genus play different roles in different contexts. The effect of *L. iners* on vaginal health may depend on specific community configuration. Genetically, this species can vary its gene expression when found within community state type (CST) IV-dominated communities (57). This intraspecific diversity may be a crucial determinant of structural stability by buffering the dominant *Lactobacillus* configuration against disturbances (58). A recent study indicated that the *L. iners* metabolite, lactate, could inhibit the proliferation and migration of cervical cancer cells (19). With respect to co-occurrence patterns, suppressed *Lactobacillus* species *L. iners* was positively associated with *Gardnerella* (59). BV-related microorganisms are more likely to be associated with HPV infection, thus, HPV infection may trigger BV establishment and promote the growth of *L. iners*, thereby promoting the progression of cervical preneoplastic lesions. However, there is little evidence regarding the potential contributors to HSIL. Similarly, one study detected a decrease in *L. crispatus* and *L. gasseri* in HPV infections, which are typically dominant in healthy vaginas (28). However, *L. crispatus* and *L. jensenii* were overrepresented in contrasting directions in HSIL and HPV infections, respectively (24, 28). Further analysis is required to determine the extent to which certain strains of *Lactobacillus* are protective or pathogenic in HPV-associated cervical diseases.

### 4.3 Confounders

Among the multiple demographic and clinical features that may account for the extensive divergences across studies, existing evidence indicates that regional and control selection are dominant. The geographical region and the correlative effect of ethnicity can influence the configuration of the vaginal microbiome (60). Our findings indicate that some of the disturbances may be specific to Asian cohorts (i.e., a differential abundance of *L. iners*). At the same time, divergence was observed in the categorization of subgroups and controls, which is problematic in disentangling the panel of vaginal microbial disturbances observed in HPV infections and cervical neoplasms. For example, Laniewski et al. (24) reported a significant increase in *L. crispatus* in HSIL versus HPV infection. However, a significant increase in *L. iners* was observed in another group compared to that in the control group (34).

Despite advancements in bioinformatics techniques in this field, vaginal microbiota studies continue to face methodological challenges. Another source of bias is methodological variations across a range of laboratory processing methods (i.e., DNA extraction, sequencing target, and platform) and data preprocessing methods (i.e., reference database and quality filtration criteria for sequences). Our analyses may also suffer from the use of different sequencing platforms as well as differences in hypervariable regions since heterogeneity in evaluating microbial diversity has been reported (60). For example, five studies reported enriched *Prevotella* in women with HPV infection using 16S rRNA gene sequencing, although a decrease was observed in one study using 16S rRNA amplicon pyrosequencing. This observation was also similar to the inconsistent changes in the taxa *Sneathia* reported by 1 study. Technical and clinical factors across studies may increase the difficulty of comparing effect sizes, indicating the need for consistency among methodologies and encouraging data sharing with sufficient metadata. The VAginaL community state typE Nearest CentroId Assifier (VALENCIA) clustering tool (12) is a novel established resolution classification program for the assignment of vaginal microbiome patterns that may be a promising step toward methodologically reinforcing consistency and reproducibility.

Other covariates, including hormonal fluctuations, contraception use, temporal activity, and HPV subtypes, are also intimately correlated with perturbations in the vaginal microbiome (23, 33, 41, 61–63). There was evidence of an association between HPV 16/18 infection and specifically increased genera, viz., *Gardnerella*, *Prevotella*, and *Atopobium* (62); however, insufficient evidence was presented in our analysis. Thus, a rigorous collection of these factors and their careful consideration in assessments and interpretation should be employed by future research groups.

### 4.4 Strengths and Limitations

To our knowledge, this is the first systematic review analyzing vaginal microbiome disturbances across a spectrum of HPV-associated diseases globally, thus forming the basis for the identification of reproducible and generalizable potential biomarkers. This enabled us to further explore its clinical use in predicting the severity of HPV-related cervical diseases as a non-invasive diagnostic tool. Functional analyses have provided new insights into the roles of specific bacteria in cervical carcinogenesis. In particular, examining the role of *Lactobacillus* species may provide better rational targets for the advancement of novel probiotic-based prevention and treatment agents. It has been shown that maintenance of the vaginal microbiota and improvement of cervical epithelialization favors regression of cervical lesions through a prebiotic effect (64). However, the present study has some limitations. First, none of the selected studies provided microbial information for cervical or cervicovaginal samples, which prevented adequate comparison of the relationship between HPV-induced cervical disease and vaginal microbiota. The decision to remove records in these samples was dictated by knowledge of the anatomic potential and substantial site-associated discrepancies in the microbiome (21, 62). Second, the geographic distribution of the included studies indicates a high proportion of studies performed in China. This circles back to the link between regional variances and vaginal bacterial clusters, an imbalance that may have affected data synthesis in our analysis. Third, most studies had relatively small or moderate sample sizes, indicating that our results may still be underpowered. Fourth, given that the selected studies varied widely in terms of study design and bioinformatic analyses, meta-analysis of the selected studies seem complicated. Therefore, we performed qualitative systematic analyses rather than a meta-analysis. Further meta-analysis based on the harmonization of methodologies may provide robust and reproducible taxonomic changes. Finally, this systematic review aimed to integrate current analyses that commonly use 16S rRNA amplicon sequencing at the horizontal taxonomic level, rather than functional profiling. The generated evidence implies that local metabolic patterns correlated with BV include amino acid, dipeptide, polyamine, and ketone body pathways (55). Recent integrative work highlighted the close association of 3-hydroxybutyrate, macrophage migration inhibitory factors, pathobionts, and dysbiotic microbiota with cervical carcinogenesis (65). Relatively little is known about the sophisticated interactive mechanisms among vaginal microbiota, metabolites, and the host. Given the recognized functional redundancy (11), harnessing multi-omics techniques will provide a window on functional candidates or metabolites to clarify the contribution of host-microbe interplay in cervical tumorigenesis.

Going forward, definitive causality for the role of specific taxa in HPV clearance and cervical diseases will stem from a more molecular-related epidemiological study at a larger scale, as well as identification of the mechanisms involved in both clinical research and experimental animal research. It is important to consider individual- and disease-related covariates using multi-omics techniques. Moreover, elucidating the potential for therapeutic manipulations of the vaginal microbiota holds promise for improving outcomes in HPV clearance and cervical lesions.

## 5 Conclusion

This systematic review reports that, in HPV infection and related cervical diseases, in addition to an increase in anaerobes, an enrichment of aerobic bacteria may characterize vaginal microbiome disturbances. *Lactobacillus iners* may play either protective or pathogenic roles in HPV infections and cervical neoplasms. Hopefully, the altered microbial taxa established in this review can pave the way for further bacterial-driven causal studies on cervical carcinogenesis.

## Data Availability Statement

The original contributions presented in the study are included in the article material. Further inquiries can be directed to the corresponding author.

## Author Contributions

FX contributed to conception and design of the review. MW conducted literature search and drafted the first manuscript. HL and HY performed the data extraction. HL, HY, YY, CW, FT and AF made substantial contributions to writing the systematic review and revising it critically. All authors contributed to manuscript revision, read, and approved the submitted version.

## Funding

This work was funded by the Tianjin Municipal Science and Technology Commission Special Foundation for Science and Technology Major Projects in Control and Prevention of Major Diseases (Grant No. 18ZXDBSY00200), the General Project of the National Natural Science Foundation of China (Grant No. 82071674), the National Natural Science Foundation of China (Grant No. 82101705), the Natural Science Foundation of Tianjin Municipal Science and Technology (Grant No. 20JCYBJC00440), the Tianjin Health Science and Technology Project (Grant No. KJ20176; KJ20003), the Scientific Research Project of Tianjin Education Commission (Grant No. 2020KJ158), and Tianjin Key Medical Discipline (Specialty) Construction Project.

## Conflict of Interest

The authors declare that the research was conducted in the absence of any commercial or financial relationships that could be construed as a potential conflict of interest.

## Publisher’s Note

All claims expressed in this article are solely those of the authors and do not necessarily represent those of their affiliated organizations, or those of the publisher, the editors and the reviewers. Any product that may be evaluated in this article, or claim that may be made by its manufacturer, is not guaranteed or endorsed by the publisher.
